# CBCT study on the prevalence, morphology and position of the mandibular incisive canal in a North-Brazilian population

**DOI:** 10.4317/jced.59487

**Published:** 2022-07-01

**Authors:** Valber-Barbosa Martins, Anne-Caroline-Costa Oenning, Luciana-Campos Guimarães, José-Luiz-Cintra Junqueira, Ademir Franco

**Affiliations:** 1 Division of Oral Radiology, Faculdade Sao Leopoldo Mandic, Brazil; 2 Division of Forensic Dentistry, Faculdade Sao Leopoldo Mandic, Brazil; 3 Centre of Forensic and Legal Medicine and Dentistry, University of Dundee, UK

## Abstract

**Background:**

The mandibular incisive canal (MIC) is an anatomic structure to be considered in treatment planning for surgeries in the anterior region of the mandible. Awareness of the MIC increased with the use of 3D imaging for treatment planning, such as cone beam computed tomography (CBCT). This study aimed to use CBCT to assess the prevalence, morphology and position of the MIC among North-Brazilians.

**Material and Methods:**

The sample consisted of CBCT scans of 100 hemi-mandibles (50 individuals) that were assessed for the absolute (n) and relative frequency of the MIC. The morphological component of this study was the diameter (mm) of the detected MIC in five anatomic sites between the mental foramen and the midline. Within the interformainal region, the position of the MIC was assessed by measuring (mm) the distances between the MIC and the basal, vestibular and lingual cortical bone surfaces.

**Results:**

The prevalence of the MIC was >76% considering the different anatomic regions screened in CBCT. The mean diameter of the MIC progressively reduced from 1.29 mm to 0.86 throughout the five anatomic regions measured. The position of the MIC showed a downward trajectory away from the lingual cortical bone surface.

**Conclusions:**

MIC was a highly prevalent anatomic structure in the studied sample. The funnel-shaped outline of the MIC and its trajectory into the interforaminal region highlighted a major risk of damage to the neurovascular bundle in surgeries (e.g. implant placement) that are close to the mental foramen and the vestibular cortical bone.

** Key words:**Anatomy, cone beam computed tomography, imaging, mandibular incisive canal, oral radiology.

## Introduction

The trigeminal nerves (fifth pair of cranial nerves – CN V) send out bilateral mandibular nerves to the lower third of the human face (1). Before entering the mandibular foramen, the mylohyoid nerve emerges, from each side, to reach and provide motricity to the mylohyoid and digastric (anterior belly) muscles (2). These muscles are fundamental to routine activities such as speaking, chewing, and swallowing (3). The sensory component of the mandibular nerve comes from the inferior alveolar nerve, located into the mandibular canal (4). Before exiting the canal through the mental foramen to become the mental nerve, the inferior alveolar nerve may send out anterior branches that continue inside the mandible as the mandibular incisive nerve (5). Together with vascular components, this bundle is lodged into the mandibular incisive canal (MIC) (6).

As with other bony structures in the maxillofacial region, the MIC may be detected in imaging exams (7) for diagnostic and therapeutic purposes, and follow-up. Proper visualization of this structure, however, may be hampered by exams that do not register the anatomical features with enough detail in the region of interest (8). This is the case of panoramic radiographs, in which the cervical vertebrae and intervertebral spaces are superimposed with the anterior teeth and bones during rotational panoramic image acquisition (9). Authors have emphasized the limitations of panoramic radiographs to enable the diagnosis of anatomic structures and variations in the anterior portion of the mandible, especially in the interforaminal region (9). Regarding the MIC, panoramic radiographs seem to underestimate (10) this structure leading to potential limitations for surgical treatment planning in dentistry and medicine (11).

Cone beam computed tomography (CBCT) Figures as an alternative (12) to visualize, measure, reconstruct, segment, and navigate through the MIC using multiplanar anatomic images. Given the cost-benefit, availability, and advanced resources of CBCT exams in the routine of maxillofacial surgery, this imaging modality represents the state-of-the-art for implant planning in the anterior region of the mandible (13). Over the last decades, populational studies on the prevalence, morphology, and anatomic position of the MIC incorporated CBCT as the imaging modality of choice (14-16).

Population-specific investigations on the MIC designed with observational-analytical methodological models are necessary to assess potential variations of these anatomic structures across different geographic samples worldwide. So far, several populations have been sampled (14-16), but there is no CBCT study on the MIC with North-Brazilians from the Amazon State. Data on this population would be beneficial to enhance the awareness of maxillofacial surgeons that operate major surgeries, including oral rehabilitation with dental implants. This study aimed to use CBCT imaging in order to assess and quantify the prevalence, morphology, and anatomic position of the MIC in a sample of North-Brazilians.

## Material and Methods

This study was performed after the approval of the local committee of ethics in human research. The Declaration of Helsinki (DoH), 2013, was followed to assure ethical standards in this medical research. The sample was collected retrospectively from a pre-existing institutional (university) database. Hence, no patient was prospectively exposed to ionizing radiation merely for research purposes. All the images that populated the database were obtained for diagnostic, therapeutic, or follow-up reasons. An observational, analytical, cross-sectional study was designed. In this context, EQUATOR (Enhancing the Quality and Transparency of Health Research) guidelines were followed and STROBE (The Strengthening the Reporting of Observational Studies in Epidemiology) checklist (17) for cross-sectional studies was used.

The sample consisted of CBCT images. The inclusion criteria set for sample collection were male and female Brazilian individuals with origin at the State of Amazonas, with at least one CBCT scan (set of images) of the full mandible taken between 2009 and 2020. Individuals should be over 14 years old (to guarantee full eruption and complete apical closure of the anterior teeth– so analyses based on the distance of teeth and the MIC could be performed).

Exclusion criteria consisted of CBCT scans missing data regarding patient’s sex, date of birth and date of image acquisition; images with visible bone lesions, or surgical appliances, and deformity of the mandible; and patients edentulous in the region between mandibular second premolars.

All the images were collected retrospectively from patients of the dentistry course, and all were obtained with diagnostic, therapeutic or follow-up purposes. Image acquisition was performed by an Oral Radiologist with the i-CAT™ unit (Imaging Science International, Hatfield, PA, USA) – energy parameters: 120 kVp, 36 mAs, exposure of 40s, and slices of 1 mm.

Image analysis was performed by a Maxillofacial Surgeon (main observer) with over 10 years of experience in the field. The observer imported the images into the Blue Sky Plan software (Libertyville, IL, USA) and used multiplanar navigation (axial, sagittal, and coronal) to assess the MIC. 3D-volume reconstructions of the CBCT scans were only used for the spatial orientation of the planes during image analysis.

**Figure 1:**
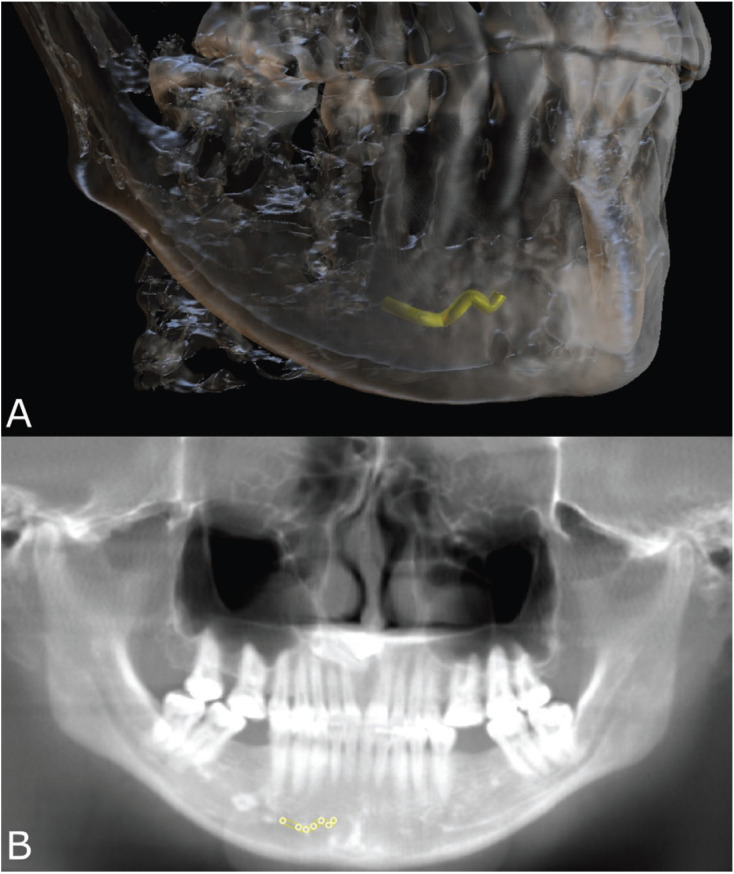
3D A) and panoramic B) reconstructions of a cone beam computed tomography volume showing the mandibular incisive canal (MIC) bone path (yellow) in the interforaminal region of the anterior portion of the mandible.

**Figure 2:**
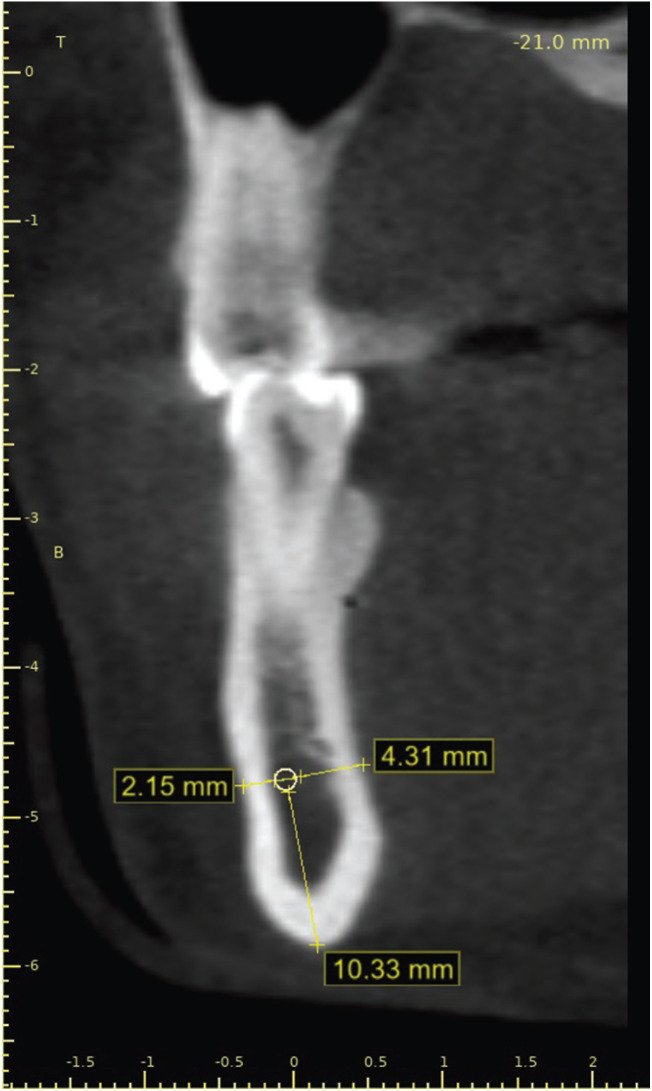
Linear measurements of the distances between the mandibular incisive canal (MIC) and the basal (10.33 mm), vestibular (2.15 mm) and lingual (4.31 mm) cortical bone surfaces.

The variables used in this study were the sex of the patient (male or female), the sides of the mandible (left or right), and the presence of the MIC (Fig. 1), its diameter (measured in different levels of the mandible, namely at initial point [5 mm medial to the mental foramen], and with increments of 3 mm, 9 mm, 12 mm and 15 mm from the initial point), and distance between the MIC and basal, vestibular and lingual cortical bone surfaces (Fig. 2).

With the mandible positioned in the Frankfurt plane, MIC measurements were performed in cross-sections perpendicular to a plane that was parallel to the basal plane of the mandible (6,18). This study considered as the MIC the hypodense path into the anterior region of the mandible (within the interforaminal space) from the mental foramen toward the median plane.

Data underwent descriptive analysis reported with absolute (n) and relative (%) values and measures of central tendency (mean and median) and dispersion (standard deviation). The measurements related to the diameter of the MIC and distances to the basal, vestibular and lingual cortical bone were quantified in millimeters (mm) and compared between males and females; compared between age categories (below or above the median age [36 years] of the sample); compared between types of patient’s dentition (partial dentition or complete set of mandibular teeth); and compared between the different level reference points of measurements (distances taking as reference the mental foramen).

Data normality was assessed with the Shapiro-Wilk test. Student’s t-tests were used to assess the effects of sex, age and location on the presence and diameter of the MIC. Analysis of variance (ANOVA) was used to assess the side-wise differences of the diameter of the MIC, and the distance of the MIC from the basal, vestibular and lingual cortical bone surfaces. Statistical significance was set at 5%, considering a confidence interval of 95%. SPSS software was used during data analysis (IBM Corp., Armonk, NY, USA).

## Results

Sample collection resulted in 50 Brazilian Amazonians (n = 17 [34%] males and 33 [66%] females) with ages between 14 and 74 years. The mean age of the sample was 38 ± 16.6 years (median = 36 years). The mean age of males was 41.2 ± 16.7 years, while in females it was 31.9 ± 15 years. In total, one-hundred hemi-mandibles were analyzed via CBCT. The mean ages between sampled males and females did not show statistically significant differences (*p* = 0.061).

When it comes to the measurements of the MIC made at the start point (5 mm from the mental foramen) and at 3 mm from the start point, all the sampled individuals (100%) presented the MIC. When the measurements were made at the level of 9mm, one right and nine left hemi-mandibles did not reveal the MIC. Hence, the prevalence of the MIC in the sample was 90% in total (90/100) – side-wise prevalence rates were 98% (49/50) and 82% (41/50) for right and left, respectively. Progressively, the diameter of the MIC decreased from 1.29 mm to 1.14 mm, 1.01 mm, 0.91 mm and 0.86 mm considering the start point, and the levels of 3 mm, 9 mm, 12 mm and 15 mm from the start point, respectively. Progressively, the distance from the MIC to the lingual cortical bone increases, while the distance to the basal and vestibular cortical bone reduces. These findings suggest that the MIC not only narrows towards the median plane but also takes a path that is more anterior and inferior in the mandible (Table 1).

**Table 1:**
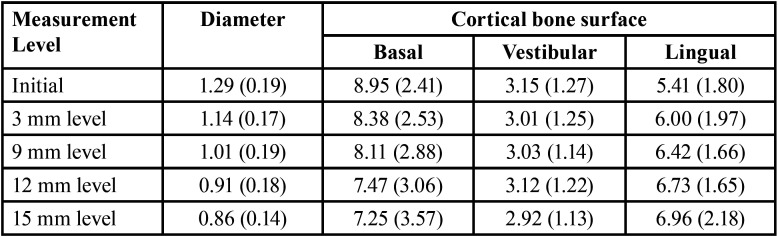
Diameter and position of the mandibular incisive canal (MIC) in the different measurement levels.

The presence of MIC changed according to the region analyzed in each image. At the level of 12 mm and the level of 15 mm from the start point, the MIC was detected in 76% and 77% of the images, respectively. The most common site to visualize the emergence of the MIC from the cortical bone was adjacent to the permanent mandibular lateral incisors (51%), followed by central incisor (36%) and canines (13%). The analysis of variance (ANOVA) showed no statistically significant differences between left and right sides for the diameter of the MIC within the different levels of measurement (*p* > 0.05), and for the distances between the MIC and the basal, vestibular, and lingual cortical bone surfaces. For this reason, future statistical analyses were performed considering a single side. Statistically significant sex differences were observed for the measurements between the MIC and basal cortical bone surface at the initial level of measurement. In males, the distances were higher than in females (*p* = 0.017). When it comes to age, the diameter of the MIC at the initial level of measurement has larger in younger individuals (≤ 36 years) compared to the older age group (> 36 years) (*p* = 0.030). For the distances between the MIC and the cortical surfaces, the only statistically significant difference was observed at the measurement level of 12 mm, in which the distance between the MIC and the lingual cortical bone surface was higher in the older age group (*p* = 0.035). Although this result was statistically significant, it was not clinically relevant since the mean distance between the MIC and the lingual cortical bone surface increased in the older age group only up to 1.19 mm. Regarding the type of dentition (complete dentition vs. partial dentition), higher distance values between the MIC and the vestibular cortical bone surface were observed (statistically significant at the measurement level of 12 mm only, *p* = 0.013). The type of dentition did not affect the diameter of the MIC significantly (*p* > 0.05) (Table 2). A deeper look at the diameter of the MIC revealed a progressive reduction from the initial measurement level to the measurement level at 3 mm (*p* < 0.05), and to the level at 9 mm (*p* < 0.05). An additional statistically significant difference was observed between the levels at 9 and 15mm (*p* < 0.05). The analysis of the position of the MIC in relation to the cortical bone surfaces revealed that longer MIC (up to 15 mm) tend to be progressively closer to the basal cortical bone surface (reducing the distance) and more distant from the lingual cortical bone surface (increasing the distance) (*p* < 0.05) (Table 3).

**Table 2:**
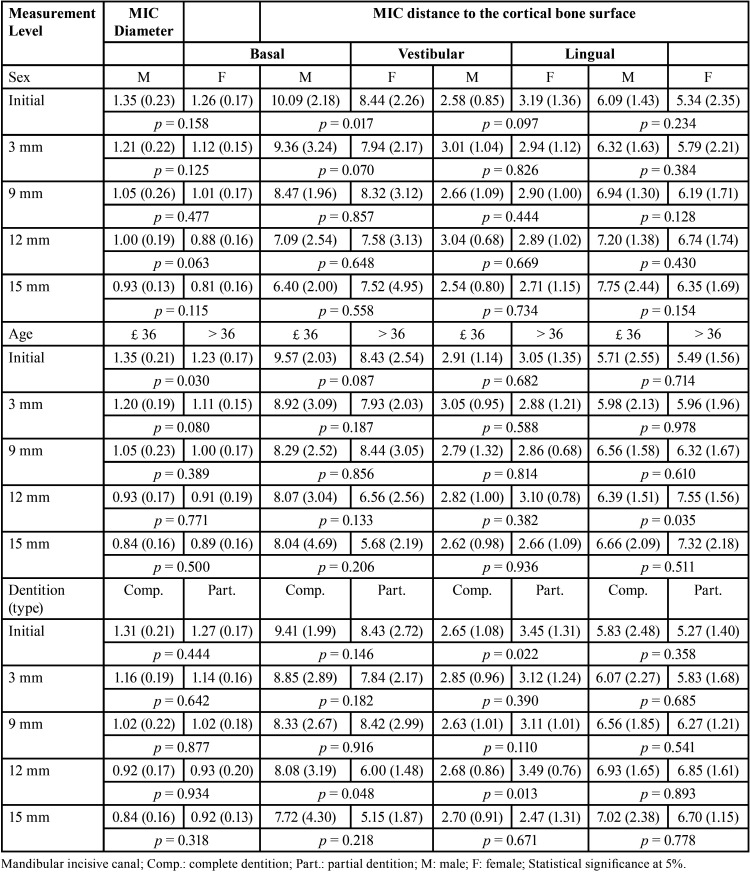
Effects of sex, age and type of dentition in the diameter of the MIC and on the distance between the MIC and the analyzed cortical bone surfaces.

**Table 3:**
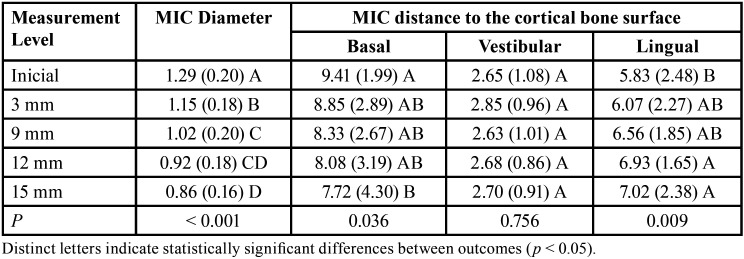
Progressive changes in the diameter of the MIC and the position of the MIC in relation to the cortical bone surfaces at each of the measurement levels established in this study (and the association between them).

## Discussion

Intercurrences in the routine of dentistry must be predicted and considered during treatment planning. From a legal perspective, dental intercurrences may be divided into accidents and complications (19). Accidents consist of an undesired event that takes place during the technical procedure, such as a broken file during endodontic treatment or a root fractured during extractions. Complications figure as postoperative events or late consequences of the treatment, such as alveolitis. The scientific literature highlights the need to consider the potential neurovascular injuries of the mandibular incisive bundle during implant surgery in the interforaminal region (20). Having in mind the importance of knowing the prevalence, morphology and position of the mandibular incisive canal, this study assessed a sample of CBCT scans from a population of the State of Amazonas, Brazil.

Dental implant placement in the anterior region of the mandible is considered a relatively minor surgical procedure (21) depending on the quantity and quality of alveolar bone present, and the systemic condition of the patient. However, considering this procedure safe, in general, denotes a lack of knowledge of the anatomic structures and variations in the mandibular region. Expecting a less complex surgery may lead to incautious implant placement planning – especially using bidimensional imaging, such as panoramic radiographs. Neglecting the existence of the MIC without proper image examination may be the first step toward malpractice. Other surgeries that involve the MIC have been reported in the scientific literature (22), such as those needed to treat bone cysts and tumors from the mandible.

This study had three main findings, sequentially related to the I) prevalence, II) morphology and III) position of the MIC. In relation to the presence of the MIC, studies are uncertain and prevalence rates reported population-specific (5,23). Over the last five years, authors have pointed out prevalence rates over 76% (not considering side-wise analyses) (5,23). The lack of solid values related to prevalence rates across populations corroborates the importance of considering the MIC prior to surgical procedures. This is to say that the possibility of finding MIC in practice should raise the awareness of surgeons and their concern towards proper treatment planning. It must be noted that detecting MIC in image exams depends on the imaging modality used (i.e. bi- or three-dimensional) and the anatomic region analyzed.

In our study, we screened the presence of MIC in five sites (initial, 3 mm, 9 mm, 12 mm and 15 mm). We observed a sequential reduction in the detection of the MIC from the initial point to 15 mm, which is explained by the fact that the MIC continues into the intraosseous path without connecting with the contralateral side in most of the cases. Benefiting from CBCT examination is fundamental to predict and control treatment effects. Authors (10) corroborate the use of CBCT for treatment planning of surgical procedures in the anterior region of the mandible by advocating that panoramic radiographs underestimate nearly 5x the detection of the MIC compared to the detection using CBCT.

Regarding the morphological component of the present study, our outcomes showed a reduced diameter of the MIC when measured at different levels progressively far from the mental foramen – starting with a mean diameter of 1.29 mm at the initial measurement point reducing to 0.86 mm at the measurement level of 15 mm far from the start point. The progressive reduction of the diameter suggests a funnel-shaped outline of the MIC and indicates that surgical intercurrences in practice may be worse if performed close to the mental foramen. Similar diameter values were observed by other authors (24) that highlighted potentially hampered osseointegration of implants placed in the MIC (due to reduced surface contact between implant and bone). The similarity between the present outcomes and the outcomes of worldwide populations (23,24) – including other Brazilian samples (6,18) shows a similar significance of the MIC in practice, which means remembering surgeons about the existing anatomic structures often neglected in treatment planning.

The distances between the MIC and the cortical bone surfaces (basal, vestibular, and lingual) were investigated as the positional component of the present study. According to our findings, the MIC was progressively closer to the basal cortical bone surface and more distant along its trajectory (statistically significant evidence was observed for this outcome). We also observed a more distant relationship with the lingual cortical bone surface compared to the vestibular surface that progressively increased from origin to terminal point. These outcomes confirm previous CBCT findings in the Brazilian population that showed a larger distance between the MIC and the lingual cortical bone surface (6).

The effects of sex and type of dentition related to the prevalence, morphology, and position of the MIC led to punctual statistically significant differences that do not necessarily represent clinical significance. In conclusion, the prevalence (%), morphology (i.e. diameter), and position (related to the basal, vestibular, and lingual cortical bone surfaces) of the MIC were investigated in the present sample of North-Brazilians using CBCT scans. Prevalence rates were above 76%. The morphological component, represented by the diameter of the MIC, reflected a funnel-shaped canal with a larger diameter in its origin decreasing towards the midline. Regarding the position, the MIC showed a downward trajectory away from the lingual cortical bone surface towards the terminal portion of the canal.
